# Ultrafast Tea Polyphenol Surface Conditioning Creates a Zincophilic Interphase for Durable Zinc Anodes

**DOI:** 10.3390/nano16160992

**Published:** 2026-08-12

**Authors:** Yimin Jiang, Chenxia Zhao, Luo Zhang, Yi Guo, Yu Jiang, Dingyu Yang

**Affiliations:** 1 Information Materials and Device Applications Key Laboratory of Sichuan Provincial Universities, Chengdu University of Information Technology, Chengdu 610225, China; 2 Optoelectronic Sensor Devices and Systems Key Laboratory of Sichuan Provincial Universities, College of Optoelectronic Engineering (Chengdu IC Valley Industrial College), Chengdu University of Information Technology, Chengdu 610225, China; 3 Chengdu Product Quality Inspection and Research Institute Co., Ltd., Chengdu 610100, China; 4 Sichuan Meteorological Optoelectronic Sensor Technology and Application Engineering Research Center, Chengdu University of Information Technology, Chengdu 610225, China

**Keywords:** aqueous zinc ion battery, zinc metal anode, tea polyphenol, surface modification, dendrite suppression, hydrogen evolution reaction

## Abstract

The practical deployment of aqueous zinc-ion batteries (AZIBs) is critically limited by uneven Zn^2+^ flux, uncontrolled dendrite growth, and concurrent parasitic reactions—notably the hydrogen evolution reaction (HER) and anode corrosion—arising from interfacial and kinetic instability during repeated plating/stripping cycles. These issues originate at the zinc anode–electrolyte interface, underscoring the necessity of advanced interfacial engineering. Here, we report a surface-confined polyphenol-derived interphase formed on zinc foil through a 1 min dip treatment in a dilute aqueous solution of a commercial tea polyphenol (TP) mixture (0.02 M); after rinsing and drying, the modified electrode is cycled in a conventional electrolyte to which no TP is deliberately added. This interphase promotes more homogeneous nucleation behaviour through coordination between phenolic oxygen-containing moieties and Zn^2+^, improves electrolyte contact homogeneity and perturbs the local water structure to mitigate water-mediated parasitic reactions. The TP-derived surface modification creates a substantially altered interfacial charging environment (Cdl = 47.25 vs. 16.83 µF cm^−2^ for bare Zn) that facilitates more uniform zinc deposition. Symmetric cells with TP@Zn anodes demonstrated exceptional cycling stability exceeding 4000 h at 1 mA cm^−2^ and 1 mAh cm^−2^ (bare Zn fails within ~240 h under identical conditions), while TP@Zn//V_2_O_5_ full cells retained 56.2% capacity after 300 cycles at 0.5 A g^−1^ with a higher median discharge voltage than bare Zn cells, substantially outperforming the latter (31.1% retention). Density functional theory calculations using the selected cluster models yield a markedly more negative electronic interaction energy for Zn^2+^ with an EGCG model ligand (−10.97 eV) than with H_2_O (−4.49 eV), qualitatively supporting preferential coordination of Zn^2+^ by phenolic oxygen sites. This work presents a green, facile and potentially scalable interfacial regulation strategy and advances the understanding of natural polyphenols as pre-formed surface conditioners for highly reversible metal anodes.

## 1. Introduction

Aqueous zinc-ion batteries (AZIBs) have emerged as a promising candidate for grid-scale energy storage due to their inherent safety, environmental benignity, and the natural abundance of zinc resources [1,2]. However, the practical deployment of AZIBs is severely hindered by the poor reversibility of the metallic zinc anode during repeated plating/stripping cycles. The instability of zinc anodes in aqueous systems mainly arises from uneven interfacial electric fields and Zn^2+^ flux, which disturb Zn nucleation and promote dendrite growth, as well as from parasitic reactions at the zinc/electrolyte interface, particularly hydrogen evolution and corrosion, which lead to irreversible zinc loss, byproduct accumulation, and a progressive increase in interfacial impedance [3,4,5,6,7,8]. Importantly, dendrite growth and HER should be understood as coupled parasitic processes originating from interfacial heterogeneity, rather than as sequential events linked by a simple cause-and-effect relationship [9,10]. Consequently, achieving highly reversible and dendrite-free zinc deposition is paramount for developing high-performance and long-life AZIBs.

Current stabilization strategies for Zn anodes can be broadly categorized into electrolyte regulation [11,12], three-dimensional zincophilic host design [13,14], advanced electrode structures [15,16], and artificial interfacial layers or surface conditioning approaches [17,18,19]. Among them, constructing an artificial solid electrolyte interphase or protective coating on the zinc anode surface has proven to be one of the most direct and effective approaches [20]. A well-designed artificial layer can physically isolate the anode from the corrosive electrolyte, homogenize the Zn^2+^ flux and electric field distribution, and thereby guide uniform zinc deposition while simultaneously suppressing detrimental side reactions [21,22,23].

Several pioneering studies have established complementary design principles for zincophilic and multifunctional protective layers on Zn anodes. A spatially gradient fluorinated alloy coating combined conductive, insulating, and three-dimensional interfacial features to promote dendrite-free Zn deposition and suppress gas evolution, while a zincophilic polyanionic hydrogel employed negatively charged-SO_3_-groups to homogenize Zn^2+^ flux and inhibit both hydrogen evolution and dendrite formation. In parallel, an In-modified anticorrosive interface demonstrated that direct suppression of the hydrogen evolution reaction can induce dense Zn deposition and markedly extend cycling life, whereas a dielectric metallic double-gradient layer coupled an electronically insulating HfO_2_ surface with a Zn-rich inner region to stabilize Zn plating/stripping at high current densities [24,25,26,27]. Collectively, these studies show that durable Zn protection requires coordinated regulation of Zn^2+^ transport and nucleation together with the suppression of interfacial water-driven parasitic reactions.

Building on these pioneering studies, recent advances in interfacial engineering have further highlighted the importance of combining Zn^2+^-affinitive regulation with control over the local electrolyte environment. For example, molecular self-assembly of hydrophobic–zincophilic bifunctional layers has delivered remarkable cycling stability [28]. Bio-derived interfacial scaffolds have also been shown to regulate Zn^2+^ electrodeposition effectively through surface engineering [29]. Despite these advances, many such systems still rely on elaborate molecular design, multi-step fabrication, or tightly controlled processing conditions, which may limit their practical scalability.

Among artificial interphases, organic and polymer-based materials have received increasing attention because of their facile processability, structural tunability, and interfacial adaptability [30]. Functional groups such as hydroxyl and carboxyl moieties can interact with Zn^2+^ and help regulate interfacial ion distribution [31]. At the same time, these materials may also perturb the local solvation or hydrogen bonding environment near the Zn surface, thereby mitigating water-mediated side reactions [9]. Compared with rigid inorganic coatings, such molecular-level interfacial regulation offers greater flexibility in balancing ion transport and side reaction suppression [32]. Nevertheless, many reported organic interphases still require complex synthesis, multi-step processing, or non-biodegradable polymer components, which raises concerns regarding cost, sustainability, and scale-up [33].

Polyphenol chemistry has already been explored for stabilizing Zn anodes through tea polyphenol electrolyte additives, tannic-acid-derived interphases and anticorrosive films, and metal–polyphenol coordination coatings [1,13,14,34]. Accordingly, the advance of this work is neither the first use of polyphenols nor the 1 min treatment by itself. Rather, it lies in the manner in which the interphase is constructed and subsequently used. A commercial tea polyphenol mixture is applied as received to Zn before cell assembly, and the treated electrode is then operated in a conventional electrolyte to which no TP is deliberately added. Specifically, a 1 min immersion in a 0.02 M aqueous TP solution produces a Zn-O-coordinated surface layer that remains detectable after rinsing and drying. This pre-formed, rinse-retained configuration separates interphase formation from cell operation and therefore distinguishes the surface-confined contribution from the continuous bulk electrolyte regulation characteristic of additive strategies. It also differs from previously reported coatings prepared from purified tannic acid or preassembled metal–polyphenol complexes [35]. Rapid layer formation is chemically plausible because catechol- and galloyl-containing polyphenols are known to assemble adherent metal-phenolic coordination networks at aqueous interfaces within short processing times [36], while tea polyphenol mixtures have been shown to chelate Zn^2+^ through oxygen-containing groups [37]. Moreover, the extensive hydration shells and hydrogen bonding capacity of green tea catechins provide a molecular basis for their interaction with interfacial water [38]. The central question addressed here is therefore whether a compositionally complex natural polyphenol feedstock can form a rapidly prepared, rinse-retained Zn interphase that simultaneously regulates Zn^2+^ deposition and attenuates water-mediated side reactions without deliberate electrolyte reformulation.

In this work, we report an ultrafast surface conditioning strategy in which zinc foil is immersed in a dilute tea polyphenol solution for one minute to form a surface-confined TP-derived interphase. The resulting interface is proposed to regulate interfacial Zn^2+^ behaviour and improve electrolyte/electrode contact homogeneity, thereby promoting more stable Zn plating/stripping while mitigating water-mediated parasitic reactions. Under 1 mA cm^−2^ and 1 mAh cm^−2^, the TP@Zn symmetric cell exhibits stable cycling for over 4000 h, whereas the bare Zn counterpart fails after about 240 h under identical conditions. When paired with a V_2_O_5_ cathode, the TP@Zn full cell also shows markedly improved cycling stability. This work therefore advances beyond a rapid coating protocol by demonstrating that a commercial, compositionally complex tea polyphenol mixture can form a Zn-O-coordinated surface layer within 1 min and that this layer remains detectable after rinsing and drying, showing that a purified single-component precursor is not essential for interphase formation. The resulting interface exhibits markedly altered charging characteristics, with a Cdl of 47.25 μF cm^−2^ compared with 16.83 μF cm^−2^ for bare Zn, together with spectroscopic changes consistent with perturbation of the interfacial water environment. These findings support a coupled interfacial mechanism in which more homogeneous Zn nucleation is accompanied by the attenuation of water-mediated side reactions. Moreover, because the interphase is constructed before cell assembly and no TP is deliberately added to the electrolyte, the observed stabilization can be attributed primarily to the surface-confined layer rather than to the continuous action of a bulk electrolyte additive.

## 2. Results and Discussion

**Figure 1:**
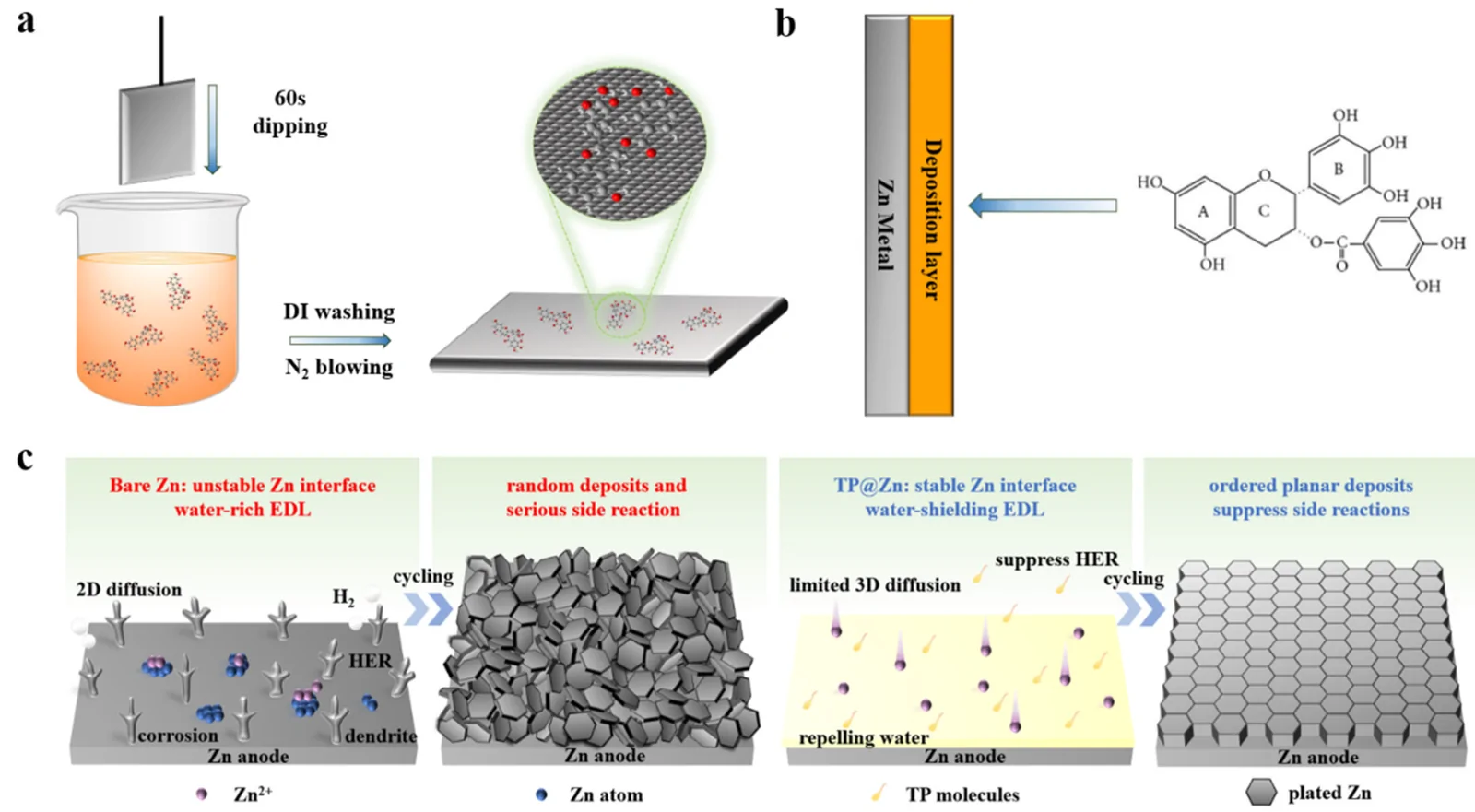
(**a**) Schematic illustration of the TP@Zn sample preparation process. (**b**) Interface configuration between electrode and electrolyte with molecular structure of TP. (**c**) Dynamic simulation of electrode surface morphology.

This study employed a simple and potentially scalable dip-coating approach, wherein zinc foils were immersed in low-concentration tea polyphenol (TP) solution for only 1 min (Figure S1). During immersion, TP molecules adsorbed effectively onto the zinc surface, forming a protective film (Figure 1a and Figure S2). Owing to the weakly acidic nature of the 0.02 M TP solution at 25 °C, partial dissociation of surface zinc oxide and zinc hydroxide occurred upon immersion, yielding Zn^2+^ ions. Concurrently, phenolic hydroxyl groups in TP underwent partial deprotonation, generating phenolate anions. The oxygen atoms of these anions, acting as electron donors, coordinated with vacant orbitals of Zn^2+^ to form Zn-O coordination bonds (Figure 1b), thereby anchoring TP molecules onto the zinc substrate. This anchoring mechanism provides interfacial protection and guides uniform zinc deposition [39,40]. In contrast, untreated bare zinc surfaces exhibited severe hydrogen evolution and parasitic reactions accompanied by intricately arranged zinc dendrites (Figure 1c).

**Figure 2:**
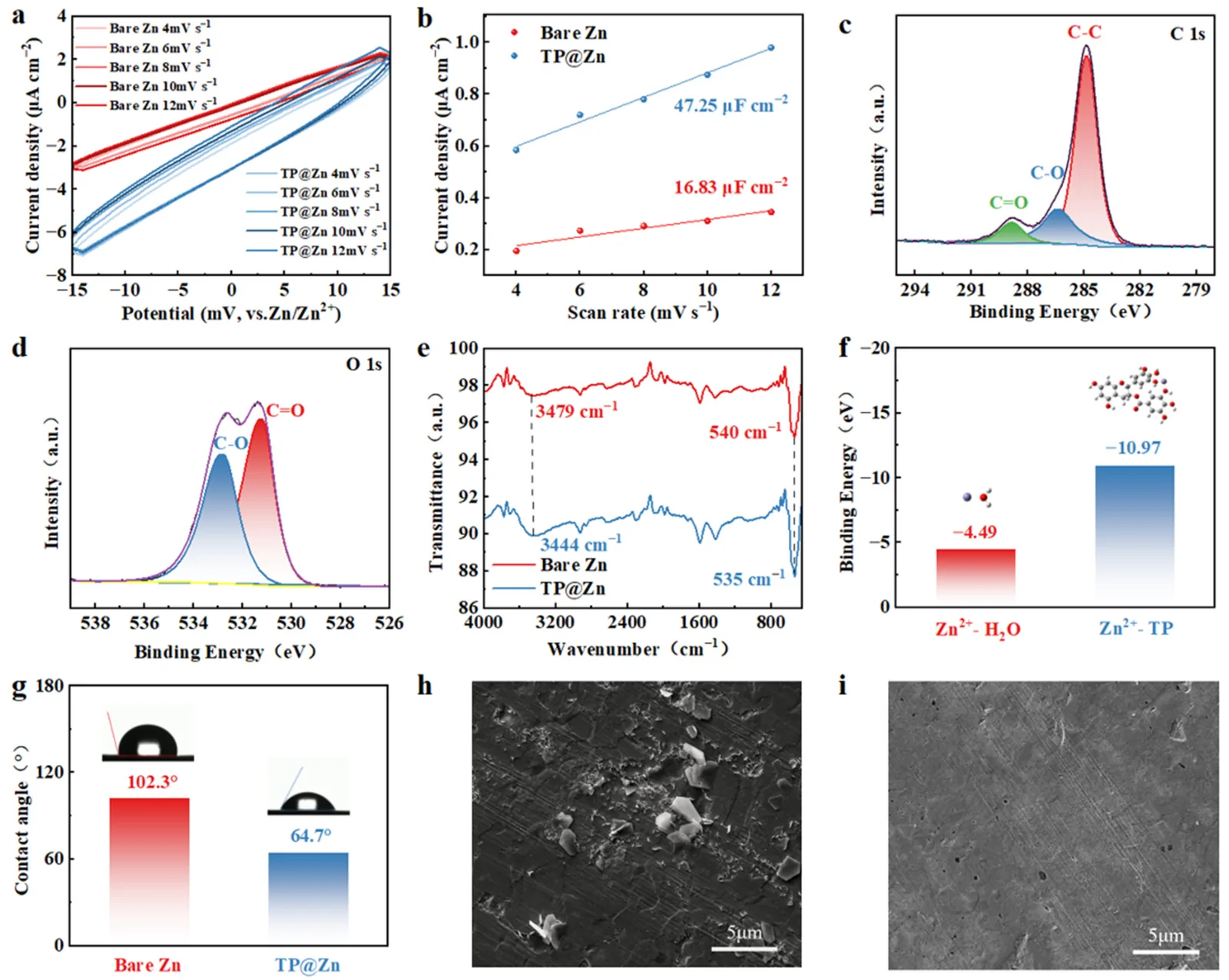
(**a**) CV profiles and (**b**) EDL plots of symmetric cells assembled with bare Zn and TP@Zn electrodes. (**c**) C 1s and (**d**) O 1s XPS spectra of TP@Zn. (**e**) FT-IR spectra comparing bare Zn and TP@Zn. (**f**) Binding energies of Zn^2+^ with H_2_O and TP molecules. (**g**) Contact angle measurements for bare Zn and TP@Zn. SEM image of (**h**) bare Zn and (**i**) TP@Zn after 2-day immersion in 2 M ZnSO_4_ electrolyte.

Cyclic voltammetry was performed on Zn//Zn cells within a voltage window of −15 to 15 mV at varying scan rates to evaluate the apparent electric double-layer capacitance at the Zn electrode surface (Figure 2a). The fitted results show that the apparent capacitance increases from 16.83 μF cm^−2^ for bare Zn to 47.25 μF cm^−2^ for TP@Zn (Figure 2b). It should be noted that electrochemically measured capacitance can be influenced by both intrinsic interfacial charging properties and the electrochemically accessible surface area associated with surface roughness or porosity [41,42]. Therefore, the approximately 2.8-fold increase cannot be attributed exclusively to altered interfacial chemistry. Although metal–polyphenol coordination is generally capable of forming conformal interfacial films [36], direct AFM measurements were not performed in the present study, and a contribution from nanoscale roughness cannot be excluded. Accordingly, the increased apparent capacitance is conservatively interpreted as evidence of an overall change in the Zn/electrolyte interfacial state after TP treatment, potentially involving contributions from both interfacial charging behaviour and effective surface area. This interfacial modification is consistent with more uniform Zn plating/stripping and improved electrochemical reversibility [43,44].

X-ray photoelectron spectroscopy (XPS) was employed to analyze the surface chemical environment of electrodes. In the C 1s spectrum, the peak at 284.8 eV corresponds to C-C bonds, attributed to aromatic ring structures in TP molecules (e.g., catechol benzene rings) (Figure 2c). This represents the core framework of TP, confirming successful adsorption of tea polyphenols onto the zinc foil surface. The peak at 286.5 eV is assigned to C-O bonds, corresponding to phenolic hydroxyl groups (-OH) in TP molecules that interact with the zinc surface as active functional groups. The weakest peak at 288.9 eV represents C=O bonds, originating from quinone (=O) or carboxyl (-COOH) groups formed through partial oxidation of TP. This indicates slight oxidation during the dip-coating process, where although partial loss of phenolic hydroxyl groups occurs, the remaining groups maintain antioxidant properties [45]. Analysis of the O 1s spectrum reveals a peak at 531 eV corresponding to C=O bonds from carbonyl groups (e.g., quinones) or carboxyl groups, consistent with the 288.9 eV peak in C 1s. The peak at 532.2 eV represents C-O bonds from phenolic hydroxyl groups (-OH) in TP molecules, matching the 286.5 eV peak in C 1s (Figure 2d) [46]. These results further demonstrate successful adsorption of TP molecules on the zinc foil surface.

Fourier transform infrared (FTIR) spectroscopy probed chemical bonding changes at the electrode–electrolyte interface (Figure 2e), providing molecular-level insights into modification mechanisms. The bare zinc battery exhibited characteristic peaks at 3479 cm^−1^ and 540 cm^−1^, corresponding to O-H and Zn-O vibrations respectively. In contrast, TP-modified cells showed peak shifts to 3444 cm^−1^ (O–H) and 535 cm^−1^ (Zn–O) with enhanced intensity. The FTIR shift together with the XPS C 1s and O 1s evolution is consistent with an altered phenolic–oxygen environment after TP treatment and, when considered alongside the comparative DFT calculations, supports the possibility of Zn–O coordination at the interface. These observations confirm strong interactions between Zn^2+^ and phenolic hydroxyl groups in TP [47,48], indicating chemical bonding interactions between TP-derived phenolic oxygen sites and the zinc surface, consistent with interfacial coordination.

Electronic interaction energies were calculated at the PBE0-D3BJ/def2-TZVP//def2-SVP level using Gaussian 16 (Figure 2f). Because commercial TP is a compositionally heterogeneous mixture without a uniquely defined molecular structure, EGCG was selected as a representative gallated–catechin ligand containing multiple phenolic oxygen donor sites. Experimental studies have demonstrated Zn(II) complexation in green tea systems [43], while NMR analysis of EGCG–metal complexes has identified the galloyl moiety as an important metal coordination region [44]. In the cluster models employed, the electronic interaction energy of Zn^2+^ with EGCG (−10.97 eV) is markedly more negative than that with H_2_O (−4.49 eV). No implicit solvation model was applied; therefore, these values represent electronic interaction energies of isolated cluster models rather than solution-phase binding free energies and are used only to establish a qualitative ordering of Zn^2+^ affinity. In the actual TP-derived interphase, multidentate or bridging coordination involving multiple phenolic groups may occur, as commonly observed in metal–polyphenol networks [36]. However, the absolute interaction energetics may vary with molecular structure, coordination geometry, protonation state, solvation environment, and computational method. Accordingly, the DFT results are interpreted only as qualitative support and are considered together with the experimental XPS and FTIR evidence for Zn-O interactions (Figure 2c–e).

Static contact angle measurements were performed on both untreated zinc foil and TP-modified zinc foil at room temperature to evaluate the hydrophilicity of zinc anode surfaces (Figure 2g). The contact angle of electrolyte on untreated zinc foil measured 102.3°, while the TP-modified surface showed a significantly reduced contact angle of 64.7°, indicating markedly improved electrolyte wettability. This hydrophilic character arises from the abundant phenolic hydroxyl (–OH) groups in TP that protrude toward the electro [16] molecules, reducing the activity of free water available for parasitic HER and corrosion reactions, an interfacial dehydration effect distinct from classical hydrophobicity.

To evaluate the corrosion inhibition capability of the TP protective layer, bare zinc foil and TP-modified zinc foil were immersed in 2 M ZnSO_4_ electrolyte for 2 days before morphological characterization using scanning electron microscopy (SEM). The bare zinc foil exhibited numerous irregular crystalline byproducts alongside pronounced surface etching cracks and heterogeneous corrosion pits (Figure 2h), demonstrating that direct electrolyte contact triggers inevitable parasitic reactions that rapidly degrade the unprotected surface. In stark contrast, the TP-modified foil maintained its original smooth morphology with only residual polishing scratches and showed no detectable corrosion or byproduct deposition (Figure 2i). The stark contrast between the severely corroded bare Zn surface and the well-preserved TP@Zn morphology after two-day immersion indicates that TP treatment provides effective surface protection against electrolyte-induced degradation. Combined with FTIR and XPS evidence for interfacial Zn–O coordination, these results support the formation of a TP-derived surface modification layer that contributes to improved corrosion resistance.

**Figure 3:**
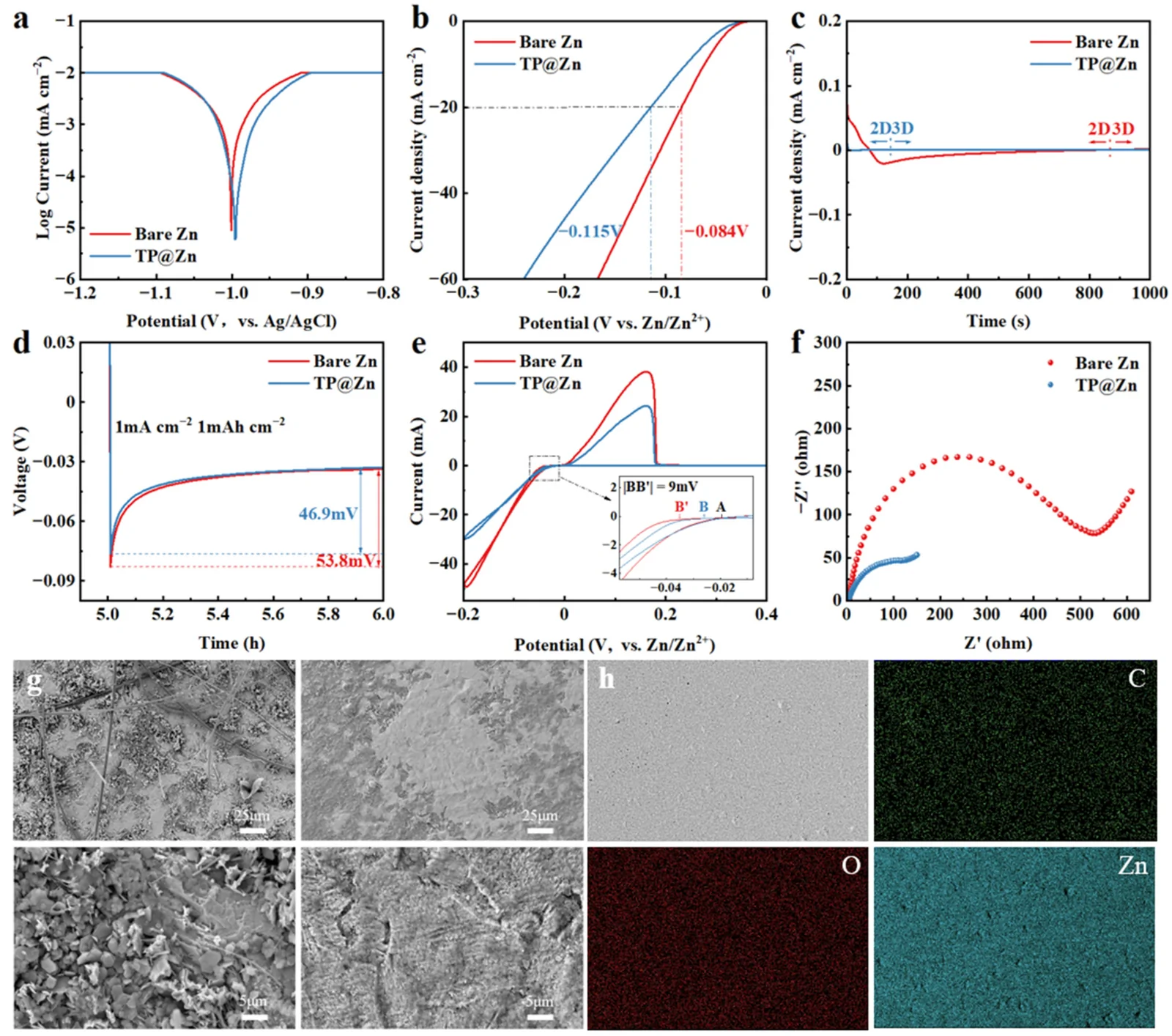
Electrochemical characterization of bare Zn and TP@Zn electrodes: (**a**) Tafel plots, (**b**) linear sweep voltammetry (LSV) curves, (**c**) chronoamperometry (CA) profiles, (**d**) nucleation overpotential, (**e**) cyclic voltammetry (CV) curves, and (**f**) Nyquist plots. (**g**) Surface SEM images of bare Zn (left) and TP@Zn (right) after 20 charge/discharge cycles at 1 mA cm^−2^ current density with 1 mAh cm^−2^ deposition capacity. (**h**) EDS elemental mapping images of TP@Zn surface, C (green), O (red), Zn (blue).

Tafel polarization analysis was performed to evaluate the corrosion kinetics and interfacial stability of the Zn electrodes (Figure 3a). The corrosion potential shifts positively from approximately −1.00 V for bare Zn to −0.98 V for TP@Zn. Meanwhile, the corrosion current density decreases from 2.24 to 1.58 mA cm^−2^, corresponding to a reduction of approximately 29.5%. The reduced corrosion current density indicates slower corrosion kinetics and suppressed parasitic interfacial reactions after TP modification, while the positive shift in corrosion potential is consistent with a reduced corrosion tendency. These results suggest that the TP coating improves the corrosion resistance and interfacial stability of the Zn anode [49].

Zn//SS cells were subsequently assembled, and linear sweep voltammetry was performed to evaluate the hydrogen evolution behaviour of bare Zn and TP@Zn (Figure 3b). TP@Zn exhibits consistently lower cathodic current densities than bare Zn at equivalent polarization potentials. At a cathodic current density of 20 mA cm^−2^, bare Zn reaches this current at −0.084 V, whereas TP@Zn requires a more negative potential of −0.115 V, corresponding to an additional polarization of approximately 31 mV. These results indicate that the TP coating inhibits HER kinetics under the tested conditions [50]. Post-cycling visual inspection of the Zn//Zn symmetric cells showed severe gas accumulation and electrolyte leakage in the bare Zn cell upon failure after approximately 240 h, whereas no visually apparent gas accumulation or electrolyte leakage was observed for the TP@Zn cell after more than 4000 h of continuous cycling (Figure S3). These macroscopic observations qualitatively support reduced gas-producing side reactions after TP modification.

Chronoamperometry (CA) analysis probed initial nucleation and surface evolution processes at the electrode interface (Figure 3c). Under a 150 mV overpotential, the control bare zinc anode required 800 s to transition from surface diffusion to three-dimensional diffusion control, indicating prolonged and irregular two-dimensional diffusion dynamics. During this phase, Zn^2+^ migrated laterally across the electrode surface, forming localized protrusions that ultimately triggered dendritic growth. In contrast, the TP-modified zinc foil entered continuous three-dimensional diffusion within merely 140 s, demonstrating rapid transition to homogeneous nucleation and planar deposition morphology. The subsequent maintenance of lower, stable current densities confirms effective suppression of Zn^2+^ surface diffusion by TP molecules [18]. This dense protective coating, formed through combined physical barrier effects and chemical adsorption, facilitates uniform zinc deposition while significantly inhibiting dendrite formation.

The nucleation overpotential was measured to evaluate the energy barrier for Zn^2+^ to overcome interfacial resistance and initiate stable deposition in Zn//Cu cells at 1 mA cm^−2^ current density with 1 mAh cm^−2^ deposition capacity (Figure 3d). TP@Zn showed a lower initial polarization (46.9 mV) compared to bare Zn (53.8 mV), indicating reduced interfacial resistance for Zn^2+^ deposition on the modified surface [51].

To investigate the influence of TP coating on zinc anode electrochemical behaviour (hydrogen evolution, zinc deposition/dissolution kinetics) and interfacial stability, Zn//Cu half-cells were assembled for cyclic voltammetry (CV) analysis of zinc stripping/deposition processes on copper substrates (Figure 3e) [52]. The TP-modified cell exhibited broader, flatter zinc dissolution peaks with reduced peak intensity, indicating more uniform and moderate dissolution that diminishes dendritic growth driving forces. Concurrently, the anodic shift in dissolution potential signifies enhanced reaction kinetics stability. Magnified views further revealed a 9 mV higher nucleation overpotential for the modified cell versus bare zinc. This elevated overpotential favours reduced critical nucleus radii [53], promoting uniform Zn^2+^ deposition across the electrode surface.

Electrochemical impedance spectroscopy (EIS) of Zn//Zn symmetric cells elucidated interfacial reaction kinetics (charge transfer resistance), surface film stability (film resistance/capacitance), and Zn^2+^ diffusion behaviour (Figure 3f), providing mechanistic insights for optimizing cycling stability and rate capability. The significantly reduced charge transfer resistance in TP-modified cells indicates accelerated deposition/dissolution kinetics. This enhancement originates from stable complexation between zinc surface Zn^2+^ ions and phenolic hydroxyl groups (-OH) in TP, which lowers activation energy barriers and facilitates charge transfer. The combined EIS and chronoamperometric results indicate that the TP interfacial layer facilitates Zn^2+^ transport at the electrode/electrolyte interface and alleviates interfacial polarization, which is favourable for regulating homogeneous Zn deposition.

To evaluate the protective effect of TP dip-coating modification on zinc anodes under practical operating conditions, Zn//Zn symmetric cells were assembled using both bare zinc foil and TP-modified zinc foil. The cells were subjected to 20 charge–discharge cycles at a current density of 1 mA cm^−2^ with a deposition capacity of 1 mAh cm^−2^ before disassembly for SEM morphological characterization. The bare zinc electrode exhibited numerous randomly distributed hexagonal zinc dendrites after cycling, whereas the TP-treated zinc foil maintained a smooth and stable surface without observable dendritic growth (Figure 3g), demonstrating the effectiveness of TP modification in promoting uniform zinc deposition and suppressing irregular dendrite formation. X-ray diffraction (XRD) analysis (Figure S4) revealed characteristic peaks of Zn_4_SO_4_(OH)_4_·0.5H_2_O on the bare zinc surface, while no corresponding peaks were detected on the TP-modified foil, confirming the suppression of parasitic reactions by the TP coating. Furthermore, post-mortem examination of separators showed distinct dendritic traces in cells with bare zinc anodes, whereas no such dendrites were observed in TP-modified cells (Figure S5), providing additional evidence for these conclusions.

Energy-dispersive spectroscopy (EDS) mapping confirmed homogeneous distribution of characteristic TP elements (carbon and oxygen) across modified surfaces, alongside uniformly dispersed zinc signals (Figure 3h). EDS mapping revealed homogeneous distribution of C and O signals across the modified Zn surface, consistent with uniform TP adsorption during dip-coating.

**Figure 4:**
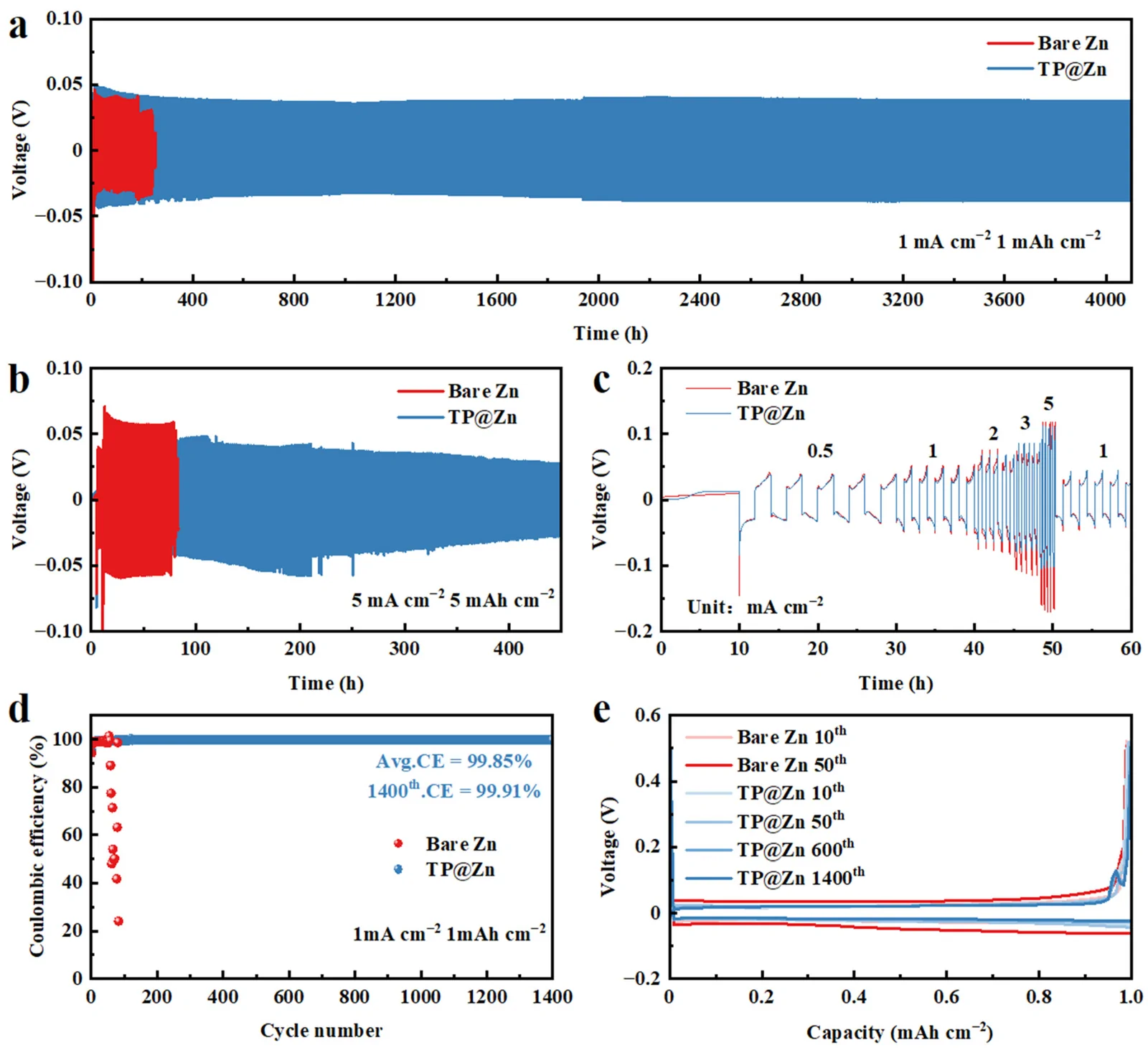
Voltage–time profiles of symmetric cells assembled with bare Zn and TP@Zn electrodes at (**a**) 1 mA cm^−2^/1 mAh cm^−2^, (**b**) 5 mA cm^−2^/5 mAh cm^−2^, and (**c**) rate capability measurements. (**d**) Coulombic efficiency and (**e**) cycling performance of Zn//Cu cells using bare Zn and TP@Zn anodes.

Cyclic stability of TP-modified and bare zinc anodes was evaluated using Zn//Zn symmetric cells. The modified cells maintained stable operation for over 4000 h at 1 mA cm^−2^ and 1 mAh cm^−2^ with consistent polarization voltages (Figure 4a). In contrast, bare zinc cells exhibited significant voltage fluctuations after 240 h before rapid failure. This degradation originates from progressively formed dendritic structures during repeated plating/stripping cycles, where excessive dendrite growth ultimately penetrated separators, causing short circuits. Remarkably, TP-modified cells achieved over 16-fold longer operational lifetimes than unmodified counterparts, demonstrating substantial cycle life enhancement. Furthermore, to determine a processing window of practical relevance, the TP concentration and immersion time were systematically screened, with the results presented in Figures S6 and S7, respectively. The screening criteria were based on the duration of stable Zn plating/stripping at 1 mA cm^−2^ and 1 mAh cm^−2^, as well as cycling stability over extended cycles. Relatively stable cycling performance was achieved across a broad range of conditions—TP concentrations from 0.01 to 0.05 M and immersion times from 0.5 to 5 min—indicating that this treatment method possesses considerable process tolerance. Among the tested conditions, 0.02 M and 1 min were ultimately selected as the standard preparation parameters, as they enable low and stable long-term polarization along with good cycling stability while minimizing both processing time and TP consumption.

The thickness of the protective interphase is also important for Zn-anode regulation. Liu et al. systematically compared nanodiamond protective layers with thicknesses of 1.6, 5.8, and 14 μm and found that the intermediate 5.8 μm layer delivered the best cycling stability, demonstrating that insufficient coverage and excessive ion transport resistance can both compromise Zn reversibility [54]. Similar considerations have recently been emphasized for natural-molecule-derived protective layers, where a non-excessive thickness is required to balance interfacial protection and Zn^2+^ transport [51]. Although the absolute thickness scale of these coated layers differs from that of our coordination interphase, the same coverage transport balance principle applies. In our case, the cycling stability remained comparably stable across the wide concentration (0.01–0.05 M) and immersion time (0.5–5 min) window rather than improving continuously with either parameter (Figures S6 and S7), indicating that the 1 min TP treatment at 0.02 M already completes the formation of a rinse-retained Zn–O-coordinated interphase. Consistently, the reduced charge transfer resistance (Figure 3f), lower nucleation overpotential (Figure 3d), and improved rate capability (Figure 4c) of TP@Zn confirm that this rapidly formed interphase provides effective interfacial regulation without impeding Zn^2+^ transport.

At higher current densities (5 mA cm^−2^/5 mAh cm^−2^), modified cells sustained approximately 450 h of stable operation, whereas bare zinc failed within 80 h due to exacerbated dendrite growth, hydrogen evolution, and corrosion (Figure 4b). This accelerated failure likely occurs when kinetic challenges such as concentration polarization, local hotspots, intensified electric fields, and rapid deposition rates exceed the protective threshold of TP layers. Subsequent localized coating compromise (e.g., penetration, cracking, or delamination) triggers explosive proliferation of dendrites and side reactions in compromised regions, rapidly degrading performance. Nevertheless, even under these demanding conditions, the TP coating still delivered significant protective effects, substantially extending battery longevity compared to unprotected anodes.

Rate capability of symmetric Zn//Zn cells was characterized under constant currents from 0.5 to 5 mA cm^−2^ (Figure 4c). TP-modified cells maintained stable cycling with consistent polarization voltages across all current densities, indicating structural integrity of the protective layer that provided uniform, low-resistance ion transport pathways. This demonstrates highly reversible zinc deposition/dissolution with exceptional reproducibility, confirming effective dendrite suppression mechanisms even at elevated rates. Conversely, bare zinc cells exhibited irregular voltage fluctuations as current densities increased. Such instability originates from inherent microstructural heterogeneity (defects, dislocations) on surfaces. At low currents, these imperfections remain benign; however, high ion fluxes rapidly amplify them into dendrites or corrosion pits, causing severe voltage fluctuations [55]. Notably, after stepwise current cycling, TP-modified cells continued stable operation beyond 2000 h at 1 mA cm^−2^, whereas bare zinc failed within approximately 160 h (Figure S8). This disparity arises because high-rate cycling irreversibly damages bare anodes through dendritic growth and parasitic reactions (hydrogen evolution/corrosion), compromising structural recovery. The exceptional resilience of modified cells directly demonstrates the ability of the TP coating to preserve electrode integrity under high-current stresses, enabling full performance recovery and long-term cyclability.

To assess electrochemical reversibility and side reactions during cycling, Zn//Cu cells were evaluated for coulombic efficiency (CE). Bare zinc cells suffered rapid CE decay within tens of cycles. In contrast, TP-modified cells sustained operation beyond 1400 cycles (Figure S9) while maintaining minimal voltage polarization (Figure S10) and an average CE of 99.85%. Remarkably, at the 1400th cycle, they persistently achieved 99.91% CE (Figure 4d), demonstrating near-complete reversibility of zinc deposition/dissolution. Zinc deposited during charging was almost entirely stripped during discharging to deliver capacity with negligible losses. Furthermore, sustained high CE implies uniform deposition without severe dendrites or “dead zinc”, suggesting TP modification promotes homogeneous nucleation and two-dimensional layered growth.

The voltage profiles of both systems directly illustrate voltage evolution during cycling (Figure 4e). Bare zinc cells showed rapid overpotential growth from 38.7 mV at the 10th cycle to 67 mV after only 50 cycles, representing a 73% rise (Figure S11). This occurs because zinc electrodes undergo continuous corrosion reactions during cycling. Byproducts constantly accumulate at the interface while disordered dendrite growth enlarges true reaction area and interfacial impedance. Significant interface deterioration appears within tens of cycles, proving inherently poor long-term stability of bare zinc. For TP-modified batteries, overpotential only changes slightly from 36.4 mV to 35 mV between the 10th and 1400th cycles, a minimal variation of 1.4 mV (Figure S12), demonstrating near-perfect stability. This conclusively verifies that the TP protective layer effectively suppresses corrosion reactions and dendrite growth, maintaining stable interface structure throughout prolonged cycling.

**Figure 5:**
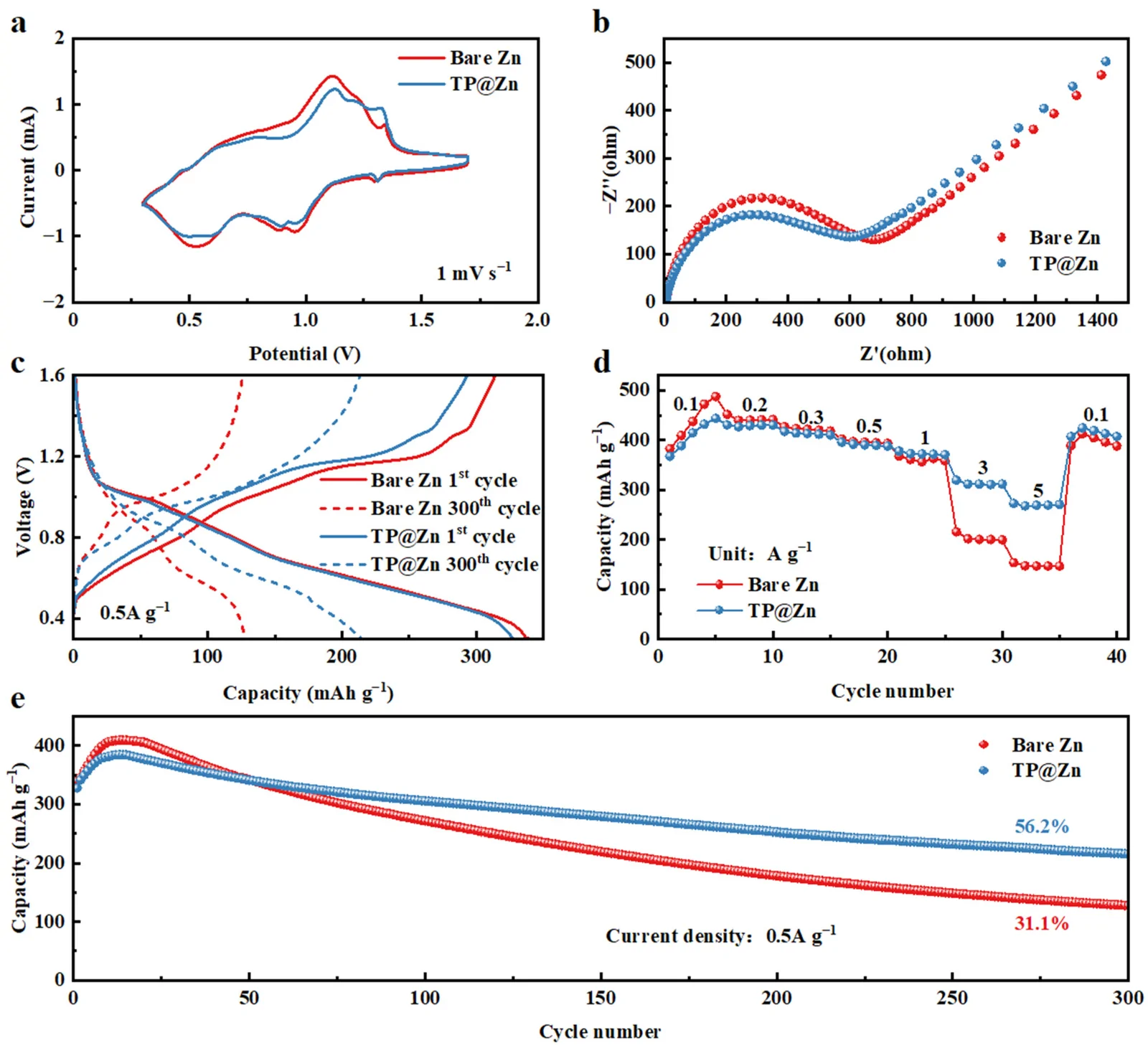
Electrochemical performance of Zn//V_2_O_5_ and TP@Zn//V_2_O_5_ full cells: (**a**) cyclic voltammetry (CV) profiles, (**b**) Nyquist plots, (**c**) galvanostatic charge–discharge (GCD) curves at 0.5 A g^−1^, (**d**) rate capability, and (**e**) capacity retention at 0.5 A g^−1^.

To further validate practical applicability of TP-modified zinc anodes, full cells were assembled with commercial V_2_O_5_ cathodes. The CV profiles of modified and bare zinc cells exhibited similar overall shapes (Figure 5a), indicating comparable electrochemical behaviour. However, the slightly smaller integrated CV area of TP-modified cells corresponds to marginally reduced initial capacity [56], potentially attributable to additional mass loading from the coating rather than active material loss. Notably, although TP-modified cells demonstrated lower maximum capacity, their capacity retention significantly outperformed bare zinc counterparts in prolonged cycling. This superior longevity stems from effective suppression of parasitic reactions by the coating, where slower capacity decay ultimately extends practical service life despite slightly diminished initial performance.

Electrochemical impedance spectroscopy (EIS) elucidated ion transport behaviour in Zn//V_2_O_5_ full cells (Figure 5b). Both samples exhibited nearly coincident high-frequency intercepts, indicating negligible differences in ohmic resistance (*R*_s_). The smaller semicircle diameter of TP-modified cells versus bare zinc confirmed reduced interfacial charge transfer resistance (*R*_ct_), owing to coordination between phenolic hydroxyl groups in TP and Zn^2+^ that lowered reaction activation energy for zinc deposition/dissolution and accelerated charge transfer. In the low-frequency region, steeper Warburg slopes for TP-modified cells demonstrated diminished Zn^2+^ diffusion impedance. This resulted from uniform TP layers guiding homogeneous zinc deposition, which prevented dendrite growth and byproduct accumulation from obstructing ion transport channels, thereby optimizing zinc-ion diffusion kinetics.

Analysis of galvanostatic charge–discharge profiles for Zn//V_2_O_5_ full cells (Figure 5c) revealed a higher initial charge plateau at approximately 1.2 V for TP-modified cells compared to 1.15 V for bare zinc, alongside smoother voltage profiles. This phenomenon arises from insufficient Zn^2+^ diffusion rates towards bare anode surfaces during charging, triggering preferential dendritic nucleation at low-overpotential sites like surface defects. Functioning as a physical barrier, the TP coating provides homogeneous nucleation sites that promote compact, dendrite-free zinc deposition rather than localized growth. Consequently, modified anodes require higher energy to overcome kinetic barriers, elevating charge plateaus. Despite marginally higher initial discharge capacity in bare zinc cells, severe degradation occurred after 300 cycles. The 300th charge curve exhibited substantial voltage drift (>0.25 V increase at 100 mAh g^−1^), indicating intensified polarization. Furthermore, discharge profiles developed steep voltage drops with vanishing plateaus and rapid capacity fading, signifying interfacial deterioration. In contrast, TP-modified cells maintained stable curve shapes with significantly mitigated capacity decay, demonstrating effective suppression of anode degradation and substantially enhanced long-term cyclability.

Rate capability evaluation at varying current densities (Figure 5d) demonstrated higher initial specific capacity for bare zinc cells at 0.1 A g^−1^, whereas TP-modified cells showed marginally reduced capacity due to coating mass loading. As current densities increased from 0.2 to 1 A g^−1^, bare cells exhibited accelerated capacity decay, contrasting with significantly slower degradation in modified cells. This confirms unhindered ion transport through TP coatings alongside effective suppression of dendrites and side reactions, thereby preserving anode integrity. At ultrahigh rates (3 A g^−1^), bare zinc capacity plunged to 200 mAh g^−1^ (44.6% decay from 1 A g^−1^). Remarkably, TP-modified cells retained 320 mAh g^−1^ (merely 16.2% decay), demonstrating superior rate tolerance. This advantage persisted at 5 A g^−1^, with modified cells outperforming bare counterparts substantially. Upon reverting to 0.1 A g^−1^, TP-modified cells recovered 424 mAh g^−1^ (95.3% retention), vastly exceeding bare zinc recovery (412 mAh g^−1^, 84.5%), confirming enhanced cycling reversibility.

Furthermore, cycling stability at 0.5 A g^−1^ revealed rapid capacity degradation to 31.1% after 300 cycles for bare zinc. TP-modified cells maintained 56.2% capacity retention (1.8-fold higher than bare zinc, representing an 80.7% improvement) with smoother decay profiles (Figure 5e). This stability arises from the dual functions of TP coatings: synergistic suppression of disordered dendrite growth through physical confinement and chemisorption, alongside mitigation of hydrogen evolution. In contrast, unprotected bare anodes suffer exacerbated side reactions and dendritic proliferation, accelerating capacity fade. Since the two full cells employed identical V_2_O_5_ cathodes and differed only in the Zn anode, the improved capacity retention of the TP@Zn//V_2_O_5_ cell is primarily associated with the enhanced reversibility and interfacial stability of the TP-modified Zn anode. This interpretation is consistent with its lower charge transfer resistance, reduced polarization growth, and improved rate recovery (Figure 5b–d). The remaining capacity decay may also involve contributions from cathode-side degradation processes commonly reported for V_2_O_5_ in aqueous Zn batteries, including cycling-induced structural evolution and vanadium dissolution [57]; notably, vanadium dissolution has likewise been identified as a key factor limiting the long-term stability of other vanadium-based cathodes, such as Li_3_V_2_(PO_4_)_3_/C composites in lithium batteries [58].

To benchmark the electrochemical performance of TP@Zn, representative natural polyphenol-based systems, artificial protective layers, and electrolyte additive strategies are compared in Table 1. Despite differences in testing conditions, TP@Zn exhibits competitive cycling stability, sustaining over 4000 h at 1 mA cm^−2^ and 1 mAh cm^−2^. Notably, this performance is achieved through a 1 min dip treatment using a commercial tea polyphenol mixture, without deliberate TP addition to the electrolyte.

## 3. Conclusions

In summary, this study proposes an ultrafast (1 min) dip-coated interfacial engineering strategy utilizing natural tea polyphenol (TP) to fabricate a surface-confined interphase on zinc anodes. Phenolic oxygen-containing moieties in TP coordinate with Zn^2+^ to promote more homogeneous nucleation behaviour and guide more uniform zinc deposition. Concurrently, the phenolic groups engage in hydrogen bonding with interfacial water molecules, perturbing the local water structure and mitigating water-mediated parasitic reactions. Under identical testing conditions (1 mA cm^−2^, 1 mAh cm^−2^), TP@Zn sustains stable cycling for over 4000 h, whereas bare Zn fails after approximately 240 h. TP@Zn//V_2_O_5_ full cells retained 56.2% capacity after 300 cycles at 0.5 A g^−1^, significantly outperforming bare zinc cells (31.1%). Density functional theory calculations at the PBE0-D3BJ/def2-TZVP//def2-SVP level using Gaussian 16 yield a markedly more negative electronic interaction energy for Zn^2+^ with an EGCG model ligand than with H_2_O in the selected cluster models, qualitatively supporting preferential coordination of Zn^2+^ by the phenolic oxygen sites of TP. This strategy offers a green and operationally simple route toward durable aqueous zinc-ion batteries. The short aqueous treatment and commercial TP mixture provide a promising basis for scale-up, with large area uniformity, processing and storage stability, TP leaching, and process-level cost representing important directions for future study.

## Figures and Tables

**Table 1:** Representative comparison of Zn-anode modification strategies reported in the literature and the present TP@Zn anode.

Strategy	Modified System	Symmetric Cell Cycling Performance	Coulombic Efficiency	Reported Polarization Metric	Representative Rate/Full Cell Performance	Ref.
Natural polyphenol electrolyte additive	Tea polyphenol additive in Zn(Ac)_2_ electrolyte	720 h at 0.5 mA cm^−2^, 0.5 mAh cm^−2^	99.78% (90 cycles, Zn‖Cu, 1 mA cm^−2^, 1 mAh cm^−2^)	~12 mV polarization (Zn‖Zn, 0.5 mA cm^−2^, 0.5 mAh cm^−2^)	Zn‖Na_3_V_2_(PO_4_)_3_: 130 mAh g^−1^ at 0.2C; 78% capacity retention after 300 cycles	[34]
Natural polyphenol interphase	Tannic-acid-derived TA@Zn	850 h at 1 mA cm^−2^, 1 mAh cm^−2^	96.8% (410 cycles, Zn‖Ti, 5 mA cm^−2^, 1 mAh cm^−2^)	57.8 mV voltage hysteresis; 76 mV initial nucleation overpotential (1 mA cm^−2^, 1 mAh cm^−2^)	TA@Zn‖V_2_O_5_: 114.4 mAh g^−1^ at 5 A g^−1^; 95.9% retention after 750 cycles at 2 A g^−1^	[39]
Metal–polyphenol coordination layer	TA-Bi-coated Zn (TBZn)	2600 h at 1 mA cm^−2^, 1 mAh cm^−2^	—	~38 mV voltage hysteresis at 1 mA cm^−2^	Stable rate response from 0.2 to 20 mA cm^−2^; improved vanadium-based (Al-VOH) full cell performance	[35]
Metal oxide protective layer	ALD-derived ZnO@Zn	680 h at 1 mA cm^−2^, 1 mAh cm^−2^	Near 100% (1000 cycles, ZnO@Zn‖Cu, 1 mA cm^−2^, 1 mAh cm^−2^)	~15.2 mV average overpotential (ZnO@Zn‖Cu, 1 mA cm^−2^, 1 mAh cm^−2^)	ZnO@Zn‖MnO_2_: 114.4 mAh g^−1^ at 5C; almost no capacity decay after 1100 cycles at 2C	[59]
Polymeric protective layer	PVDF-HFP-coated Zn (P-Zn)	700 h at 1 mA cm^−2^, 1 mAh cm^−2^	~100% (~1500 cycles, P-Zn‖Cu, 1 mA cm^−2^, 1 mAh cm^−2^)	—	P-Zn‖MnO_2_: retained nearly full capacity after 1400 cycles at 2C	[60]
Carbon-based protective layer	N-doped reduced-graphene-coated Zn (N-rGO@Zn)	>1200 h at 1 mA cm^−2^, 1 mAh cm^−2^	98.9% (Cu‖N-rGO@Zn, 1 mA cm^−2^, 0.5 mAh cm^−2^)	42.1 mV voltage hysteresis (1 mA cm^−2^, 1 mAh cm^−2^)	N-rGO@Zn‖MnVO: 151.0 mAh g^−1^ at 10 A g^−1^; 97.0% retention after 2500 cycles	[61]
This work	Tea polyphenol dip-coated Zn (TP@Zn, 0.02 M, 1 min)	>4000 h at 1 mA cm^−2^ and 1 mAh cm^−2^	99.85% average; 99.91% at 1400th cycle (Zn‖Cu, 1 mA cm^−2^, 1 mAh cm^−2^)	~35–36 mV stable voltage hysteresis (1 mA cm^−2^, 1 mAh cm^−2^); 46.9 mV nucleation overpotential	TP@Zn‖V_2_O_5_: 56.2% capacity retention after 300 cycles at 0.5 A g^−1^ (bare Zn 31.1%)	

## Data Availability

The original contributions presented in this study are included in the article/Supplementary Materials. Further inquiries can be directed to the corresponding authors.

## Supplementary Materials

The following supporting information can be downloaded at: https://www.mdpi.com/article/10.3390/nano16160992/s1, Figure S1: Photographs of 0.01, 0.02, and 0.05 M TP solutions; Figure S2: Optical images of bare Zn and TP@Zn foils; Figure S3: Comparative optical images of bare Zn and TP@Zn symmetric cells after long-term cycling; Figure S4: XRD pattern of pristine Zn, bare Zn and TP@Zn after 20 cycles (1 mA cm^−2^, 1 mAh cm^−2^); Figure S5: Separator from bare Zn//Zn and TP@Zn//Zn cell after 20 cycles (1 mA cm^−2^, 1 mAh cm^−2^); Figure S6: Long-term cycling stability of Zn symmetric cells in tea polyphenol solutions with different concentrations (0.01–0.05 M) at 1 mA cm^−2^ and 1 mAh cm^−2^; Figure S7: Long-term cycling stability of Zn symmetric cells at 1 mA cm^−2^, 1mAh cm^−2^ by different soaking time (0.5–5 min); Figure S8: Cycling stability of rate-cycled bare Zn and TP@Zn symmetric cells (0.5→1→2→3→5→1 mA cm^−2^); Figure S9: Voltage profiles of the Zn//Cu and TP@Zn//Cu half-cells during cycling; Figure S10: Voltage profiles of the Zn//Cu and TP@Zn//Cu half-cells during partial cycling; Figure S11: Voltage profiles of the Zn//Cu asymmetric cells at different cycles at 1 mA cm^−2^ and 1 mAh cm^−2^; Figure S12: Voltage profiles of the TP@Zn//Cu asymmetric cells at different cycles at 1 mA cm^−2^ and 1 mAh cm^−2^.
