# Supplementary material for: Ultrafast Tea Polyphenol Surface Conditioning Creates a Zincophilic Interphase for Durable Zinc Anodes
Source: Nanomaterials (Basel). 2026 Aug 12;16(16):992. doi: 10.3390/nano16160992 (PMC13516990; doi:10.3390/nano16160992)
Supplement: Supplementary file 1 [file nanomaterials-16-00992-s001.zip › nanomaterials-4474345-supplementary.pdf]

Supporting Information

Ultrafast Tea-Polyphenol Surface Conditioning Creates a Zincophilic Interphase for Durable Zinc Anodes

Yimin Jiang[a#], Chenxia Zhao[a#], Luo Zhang[a], Yi Guo[b], Yu Jiang[*c], Dingyu Yang[*d]

a *Information Materials and Device Applications Key Laboratory of Sichuan Provincial Universities, Chengdu University of Information Technology, Chengdu 610225, PR China*

b *Optoelectronic Sensor Devices and Systems Key Laboratory of Sichuan Provincial Universities, College of Op-toelectronic Engineering (Chengdu IC Valley Industrial College), Chengdu University of Information Technology, Chengdu 610225, PR China*

c *Chengdu Product Quality Inspection and Research Institute Co., Ltd, Chengdu 610100, PR China*

d *Sichuan Meteorological Optoelectronic Sensor Technology and Application Engineering Research Center, Chengdu University of Information Technology, Chengdu 610225, PR China*

* Correspondence: *jiangyu@cqi.org; yangdingyu@cuit.edu.cn*

\# Yimin Jiang and Chenxia Zhao contributed equally to the work and should be considered co-first authors.

Experimental Section

S1. Material preparation

$ZnSO_4 \cdot 7H_2O$ (analytical grade) was purchased from Kelong (Chengdu, China). Zinc foil (thickness: 0.1 mm), copper foil (thickness: 0.1 mm), CR2016 coin-cell components, and glass fiber separators (Whatman GF/D, diameter: 25 mm) and commercial vanadium pentoxide (V_2O_5) cathode with an active material loading of 0.98 mg cm^{-2} were purchased from Kelude (Dongguan,

China). Anhydrous ethanol (≥99% purity) was purchased from Aladdin (Shanghai, China). Deionized water (18.2 MΩ·cm resistivity) was self-made in the laboratory.

The electrolyte was prepared by dissolving 23.0064 g $ZnSO_4 \cdot 7H_2O$ in 40 mL deionized water under magnetic stirring for 2 h at ambient temperature, yielding a 2 M aqueous solution. Zinc anodes were categorized into bare and TP-modified groups, both fabricated from 100 μm zinc foil substrates. All foils underwent sequential 15-min ultrasonication in deionized water and ethanol to remove surface contaminants, followed by vacuum drying at 60 °C for 2 h and punching into 14 mm diameter disks. For TP-modified anodes, 0.9168 g tea polyphenol was dissolved in 100 mL deionized water (0.02 M) with 15-min stirring. The prepared zinc foil was immersed in the tea polyphenol solution for one minute before being carefully removed. The modified foil was subsequently rinsed with deionised water followed by anhydrous ethanol, with all residual moisture being removed under a nitrogen stream. The sample was then dried in a vacuum oven at 60°C for two hours, yielding the final treated zinc anode for experimental use. Copper electrodes were prepared by ethanol-wiping, air-drying and punching into 14 mm disks. Coin cells were assembled using 19 mm diameter electrodes and separators. Each cell was precisely dripped with 75 μL electrolyte via micropipette.

S2. Material characterization

Scanning electron microscope (SEM, Hitachi SU8020) was used to characterize the surface morphology of zinc foil. The surface composition of the samples has been confirmed by X-ray diffraction (XRD, Bruker D8 Advance) in the scan range of $2\theta = 10°$ to 90°, the scanning speed was 0.5° min^{-1}. The surface chemistry of zinc foil was characterised by X-ray photoelectron spectroscopy (XPS, Thermo Scientific K-Alpha). The evolution of chemical bonds at the interface between the electrode and the electrolyte was detected using Fourier transform infrared spectroscopy (FTIR, Thermo Fisher Scientific Nicolet iS20).

The wettability of the negative electrode with the electrolyte was tested with a contact angle tester (Dataphysics OCA 20).

S3. Electrochemical tests

Galvanostatic charge/discharge (GCD) and long-term stability were tested on a NEWARE multi-channel workstation (MIHW-200-160CH-B, China) at 25°C. The symmetric cells underwent long-term cycling at 1 and 5 mA cm^{-2} with fixed areal capacities of 1 and 5 mAh cm^{-2} respectively. Full cells were evaluated for rate capability (0 - 5 A g^{-1}) and cycling stability (0.5 A g^{-1}). Tafel plots were measured using metallic zinc as the working electrode, a platinum electrode as the counter electrode, and a saturated calomel electrode (SCE) as the reference electrode, with Tafel polarization scans from -1.2 to -0.3 V at 1 mV s^{-1}. Linear sweep voltammetry (LSV) was performed in two-electrode Zn//stainless steel (SS) coin cells at the scan rate of 1 mV s^{-1}. EIS data were recorded between 0.01 and 10^5 Hz at an amplitude of 10 mV. Tafel plots, linear sweep voltammetry (LSV), chronoamperometry (CA), cyclic voltammetry (CV), double-layer capacitance (C_{dl}) and electrochemical impedance spectroscopy (EIS) were measured using an electrochemical workstation (CHI 660E, CH Instruments, China). All electrochemical tests were performed at room temperature.

S4. Computational methods

All calculations were performed using the Gaussian 16 software package. Geometrical optimisations of all species employed the def2-SVP basis set, whilst high-accuracy single-point energy calculations utilised the def2-TZVP basis set with the PBE0-D3BJ functional. The spin multiplicity was set to 1 (singlet state) without symmetry constraints or additional empirical parameters. Monomeric molecular models were constructed and fully optimised at this computational level. Subsequent single-point energy calculations were executed on the optimised structures under identical theoretical conditions.No implicit solvation model was employed in these calculations. Therefore, the reported values are electronic interaction energies of the cluster models rather than solution-phase

binding free energies and are used in the main text only for qualitative comparison.

Supplementary Figures

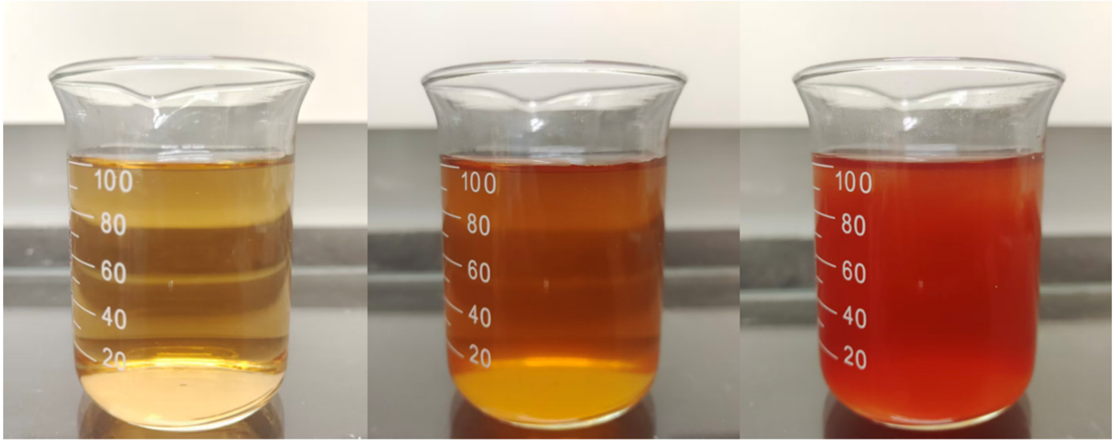

Figure S1 Photographs of 0.01, 0.02, and 0.05 M TP solutions.

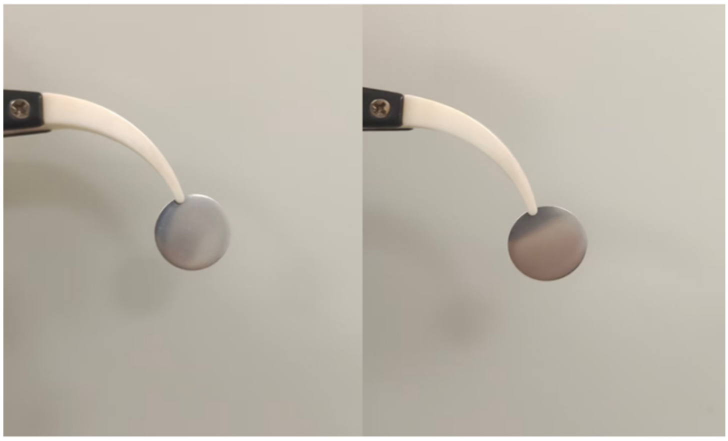

Figure S2 Optical images of bare Zn and TP@Zn foils.

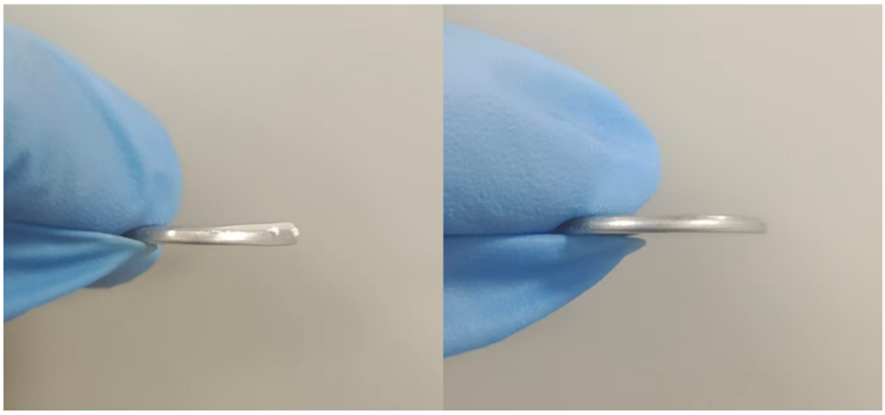

Figure S3 Comparative optical images of bare Zn and TP@Zn symmetric cells after long-term cycling.

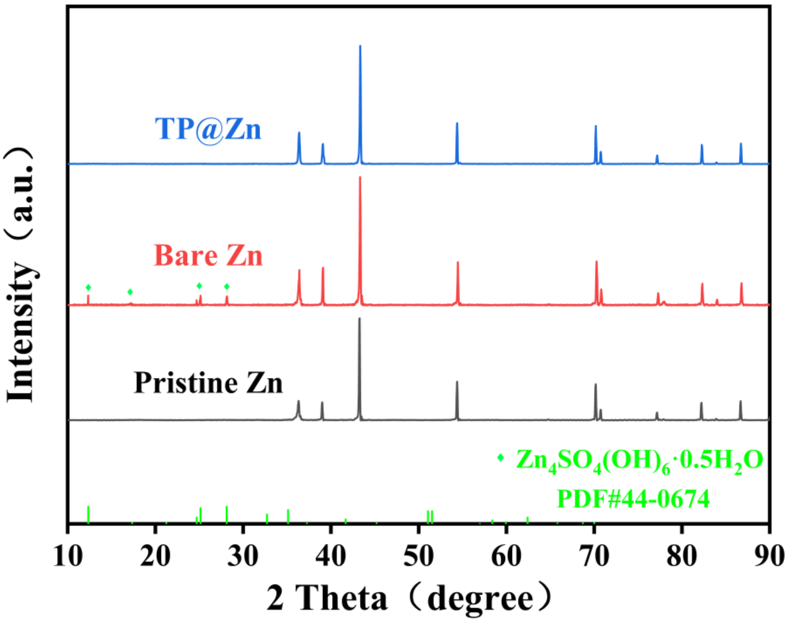

Figure S4 XRD pattern of pristine Zn, bare Zn and TP@Zn after 20 cycles (1 mA cm^{-2}, 1 mAh cm^{-2}).

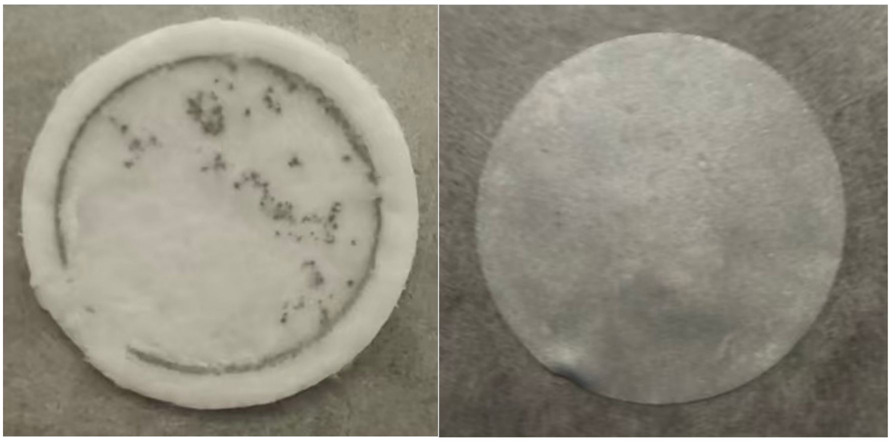

Figure S5 Separator from bare Zn//Zn and TP@Zn//Zn cell after 20 cycles (1 mA cm^{-2}, 1 mAh cm^{-2}).

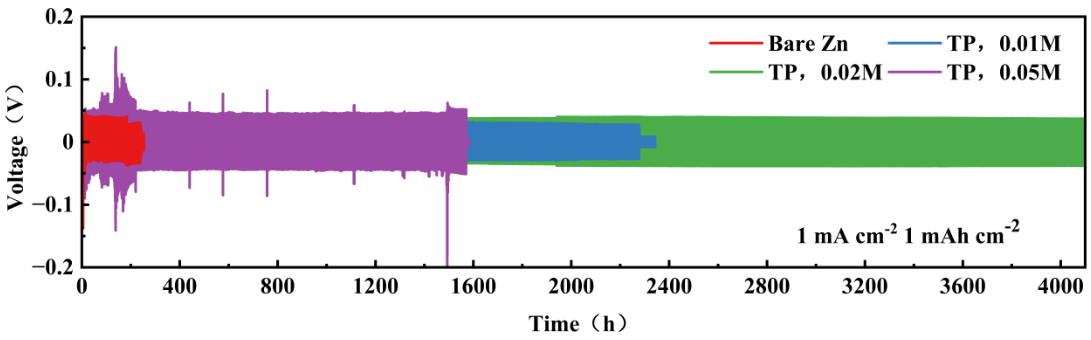

Figure S6 Long-term cycling stability of Zn symmetric cells in tea polyphenol solutions with different concentrations (0.01–0.05 M) at 1 mA cm^{-2} and 1 mAh cm^{-2}.

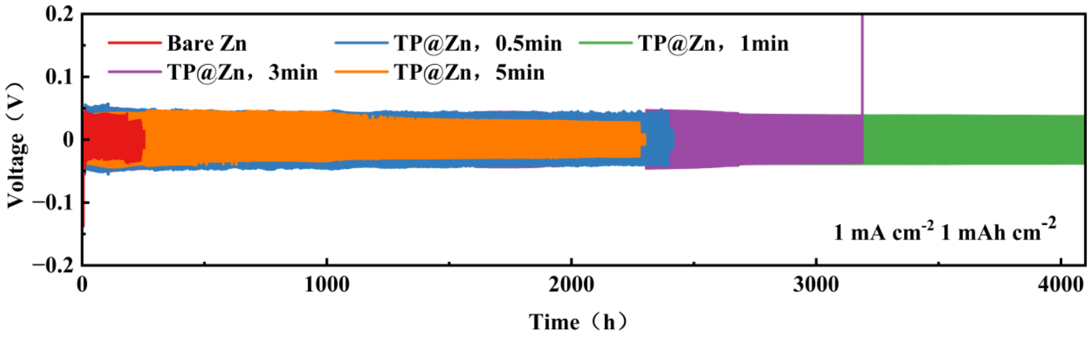

Figure S7 Long-term cycling stability of Zn symmetric cells at 1 mA cm^{-2}, 1mAh cm^{-2} by different soaking time (0.5 – 5 min).

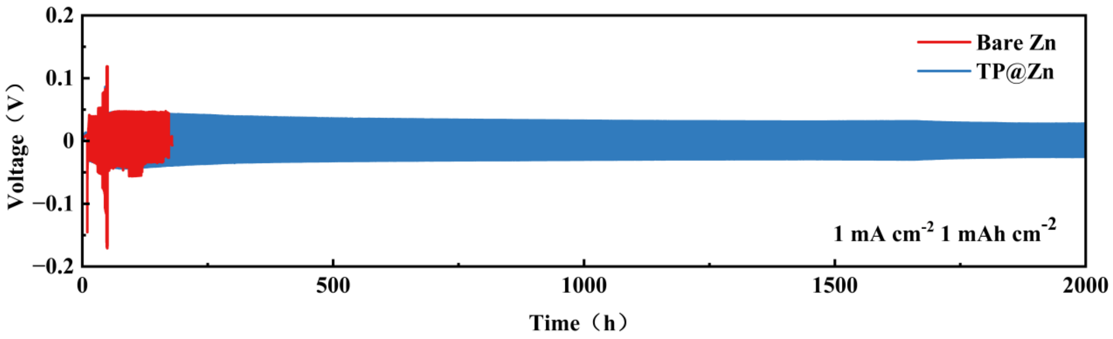

Figure S8 Cycling stability of rate-cycled bare Zn and TP@Zn symmetric cells (0.5 →1→2→3→5→1 mA cm^{-2}).

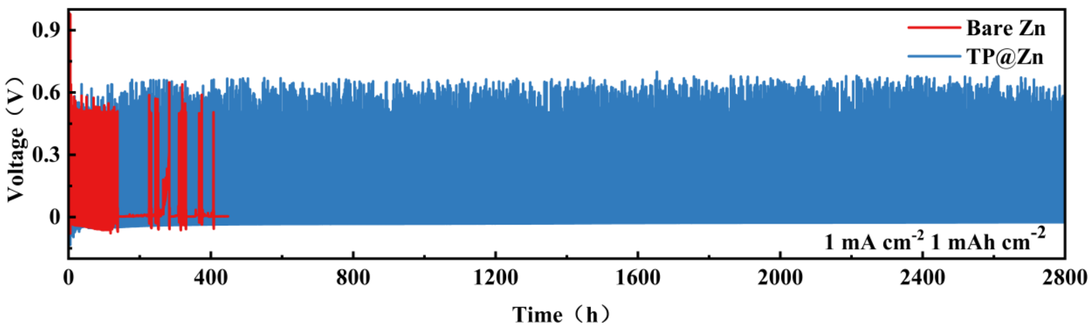

Figure S9 Voltage profiles of the Zn//Cu and TP@Zn//Cu half-cells during cycling.

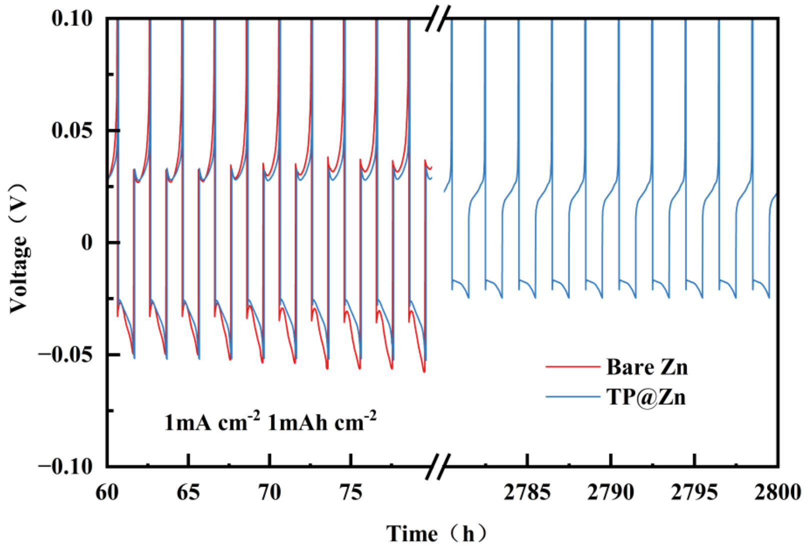

Figure S10 Voltage profiles of the Zn//Cu and TP@Zn//Cu half-cells during partial cycling.

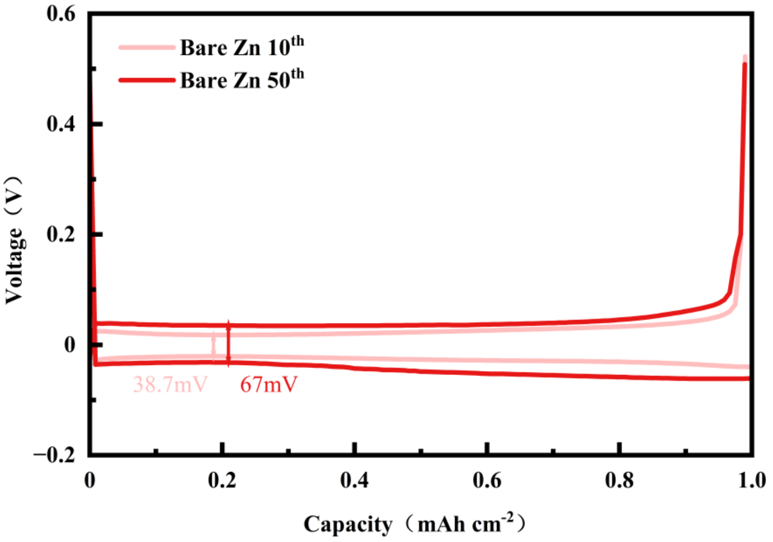

Figure S11 Voltage profiles of the Zn//Cu asymmetric cells at different cycles at 1 mA cm^{-2} and 1 mAh cm^{-2}.

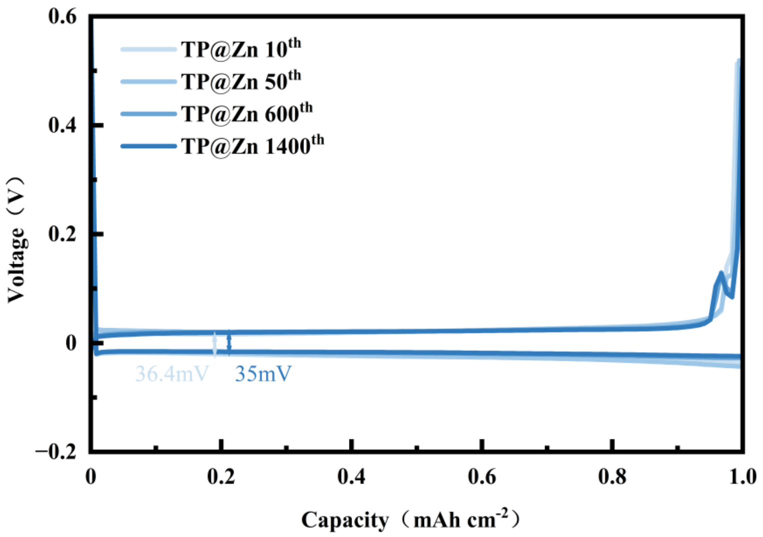

Figure S12 Voltage profiles of the TP@Zn//Cu asymmetric cells at different cycles at 1 mA cm^{-2} and 1 mAh cm^{-2}.
